# Highly Sensitive Photoacoustic Microcavity Gas Sensor for Leak Detection

**DOI:** 10.3390/s20041164

**Published:** 2020-02-20

**Authors:** Ke Chen, Yewei Chen, Bo Zhang, Liang Mei, Min Guo, Hong Deng, Shuai Liu, Fengxiang Ma, Zhenfeng Gong, Qingxu Yu

**Affiliations:** 1 School of Optoelectronic Engineering and Instrumentation Science, Dalian University of Technology, Dalian 116024, China; wxcyw@mail.dlut.edu.cn (Y.C.); zhangbobo@mail.dlut.edu.cn (B.Z.); meiliang@dlut.edu.cn (L.M.); gmduter@mail.dlut.edu.cn (M.G.); june@mail.dlut.edu.cn (H.D.); liushuaiyes@mail.dlut.edu.cn (S.L.); gongzf@dlut.edu.cn (Z.G.); yuqx@dlut.edu.cn (Q.Y.); 2 Electric Power Research Institute of State Grid Anhui Electric Power Co., Ltd., Hefei 230601, China; njumfx@foxmail.com

**Keywords:** gas leak detection, photoacoustic spectroscopy, microcavity gas sensor, gas diffusion, wavelength modulation spectroscopy

## Abstract

A highly sensitive photoacoustic (PA) microcavity gas sensor for leak detection is proposed. The miniature and low-cost gas sensor mainly consisted of a micro-electro-mechanical system (MEMS) microphone and a stainless-steel capillary with two small holes opened on the side wall. Different from traditional PA sensors, the designed low-power sensor had no gas valves and pumps. Gas could diffuse into the stainless-steel PA microcavity from two holes. The volume of the cavity in the sensor was only 7.9 μL. We use a 1650.96 nm distributed feedback (DFB) laser and the second-harmonic wavelength modulation spectroscopy (2*f*-WMS) method to measure PA signals. The measurement result of diffused methane (CH_4_) gas shows a response time of 5.8 s and a recovery time of 5.2 s. The detection limit was achieved at 1.7 ppm with a 1-s lock-in integral time. In addition, the calculated normalized noise equivalent absorption (NNEA) coefficient was 1.2 × 10^−8^ W·cm^−1^·Hz^−1/2^. The designed PA microcavity sensor can be used for the early warning of gas leakage.

## 1. Introduction

Gas leak detection plays an important role in the safety monitoring of gas pipelines, chemical plants, coal mines, and residential buildings [1,2,3,4,5]. The leakage of flammable or explosive gases, such as natural gas, can cause serious safety incidents. Trace diffuse gas detection provides an effective method for the early warning of these accidents.

Traditional methods for gas leak detection mainly include electrochemical sensing, semiconductor sensing, and tunable diode laser absorption spectroscopy (TDLAS). In recent years, electrochemical gas sensors and semiconductor gas sensors have been widely used for their low cost and small size [6,7]. However, they both have poor gas selectivity, and the former even has a short working life. In addition, if the semiconductor sensor is not sustained in the probe gas for a long time, it will enter the sleep state due to oxidation. The TDLAS-based optical measurement method can effectively solve the problems of a short working life and poor gas selectivity [8,9,10]. However, the single-pass absorption cell based TDLAS system cannot simultaneously improve the gas detection sensitivity and reduce the volume of the gas chamber. This is because the detection sensitivity is proportional to the absorption path length. As a result, a huge single-pass or a complex multi-pass absorption cell is required to improve the detection sensitivity to the ppm-level. However, the volume of the gas cell in a high-sensitivity TDLAS system is usually greater than 1 L.

Due to the characteristics of almost background-free absorption, photoacoustic (PA) spectroscopy (PAS) is one of the most sensitive trace gas detection methods [11,12,13,14,15,16]. A particular advantage is that the detection sensitivity is generally inversely proportional to the volume of the PA cell, which can effectively reduce the volume of the absorption cell [17,18]. In recent years, various PA gas sensing systems have been proposed for trace CH_4_ detection. Sampaolo et al. presented a compact quartz-enhanced PAS (QEPAS) sensor prototype for trace gas detection of CH_4_, C_2_H_6_, and C_3_H_8_ by using a mid-infrared interband cascade laser (ICL) operating in the spectral range 3.342–3.349 μm [19]. A minimum detection limit (MDL) of 90 ppb for CH_4_ was obtained with a 1-s integration time. However, due to the small gap between the two tuning fork prongs, the requirement for the quality of the light beam is very high. The non-resonant PA cell with an acoustic sensor (e.g., a cantilever) can also be used for trace CH_4_ detection. Peltola et al. demonstrated a cantilever-enhanced PAS (CEPAS) based CH_4_ detection system by utilizing a mid-infrared tunable optical parametric oscillator (OPO) [20]. The MDL achieved 65 ppt with a 30-s averaging time. However, gas valves and pumps, which are usually required in non-resonant PAS systems, need to be closed during the measurement. Therefore, continuous gas leak monitoring is impossible in non-resonant PAS systems. In order to improve the detection sensitivity and achieve the flow measurement, a variety of resonant PA cells with buffers were used. Zheng et al. used a 22-stage mid-infrared interband cascade light emitting device (ICLED) with an optical power of ~700 μW to emit excitation light into a differential resonant PA cell to detect CH_4_ [21]. When the integral time was 1 s, the assembled system showed that the MDL was ~3.6 ppm. To reduce the cost of the light source, near-infrared lasers with a wavelength around 1650 nm were utilized by Wang et al. [22] and Mao et al. [23], successively. The MDLs reached 10 ppm and 64 ppb, respectively. However, the volumes of the H-type resonant PA cells were usually larger than 200 mL. Moreover, air pumps were still required.

In this paper, we propose a highly sensitive PA microcavity sensor without gas valves and pumps for leak detection. The low-power and low-cost PA gas sensor mainly consists of a micro-electro-mechanical system (MEMS) microphone and a stainless-steel capillary with two small holes opened on the side wall. The leaking gas diffuses into the stainless-steel PA microcavity from two holes. We use the second-harmonic wavelength modulation spectroscopy (2*f*-WMS) method to measure PA signals. The optimized design method for the sensor is provided. Furthermore, the developed PA gas sensor is tested by detecting the diffusing CH_4_ gas.

## 2. Design of the Sensing System

### 2.1. Design of the PA Microcavity Sensor

**Figure 1:**
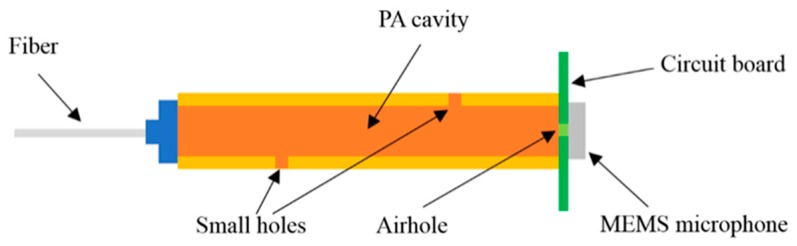
Structure diagram of the photoacoustic (PA) microcavity sensor.

The structure diagram of the PA microcavity sensor is shown in Figure 1. A single-mode fiber, a stainless-steel capillary, and a MEMS microphone mainly form the gas sensing head. From the two small holes opened by a laser marker on the side wall of the stainless-steel capillary, gas can diffuse into the stainless-steel PA microcavity. The modulated laser light is injected into the microcavity through the single-mode fiber to excite the PA pressure wave, which is detected by the MEMS microphone (ICS-40730, Invensense) with a sound sensitivity of 25 mV/Pa and a relatively flat frequency response from 25 Hz to 25 kHz.

In an unsealed cylindrical PA cavity, the generated PA pressure can be expressed as [24]:

(1) $$P_{\mathrm{PA}}\left(\omega \right)=-\frac{i\omega }{\omega_{j}^{2}\left[1-{\left(\frac{\omega }{\omega_{j}}\right)}^{2}-i\frac{\omega }{\omega_{j}Q_{j}}\right]}\frac{\alpha (\gamma -1)P_{0}}{\pi r^{2}}H(\tau_{1})H(\tau_{2})$$

(2) $$H(\tau_{1})=\frac{\tau_{1}}{\sqrt{1+{\left(\omega \tau_{1}\right)}^{2}}}$$

(3) $$H(\tau_{2})=\frac{\omega \tau_{2}}{\sqrt{1+{\left(\omega \tau_{2}\right)}^{2}}}$$

where *ω* is the modulation angular frequency, *ω_j_* is the *j*th order resonance angular frequency of the PA cavity, *Q_j_* is the acoustic resonance quality factor at *ω_j_*, *α* is the gas absorption coefficient, *r* is the inner radius of the PA microcavity, *P*_0_ is the light power, *γ* is the specific heat ratio, *τ*_1_ is the thermal relaxation time, and *τ*_2_ is the relaxation time generated by gas and heat flow through the small holes. *τ*_1_ and *τ*_2_ can be expressed as:

(4) $$\tau_{1}=\frac{r^{2}}{5.78D_{T}}$$

(5) $$\tau_{2}=\frac{4\sqrt{\gamma }V}{3\upsilon A_{g}}$$

where *D*_T_ is the gas thermal diffusivity, *υ* is the sound velocity in the PA cavity, and *A*_g_ is the total area of the gas diffuse holes.

According to Equations (1)–(5), the magnitude of the generated PA pressure is nearly inversely proportional to the square of the inner radius of the PA cavity. To reduce the size of the PA cavity as much as possible to shorten the response time without increasing the processing difficulty, the inner radius of the stainless-steel cylindrical cavity was set to 0.5 mm.

**Figure 2:**
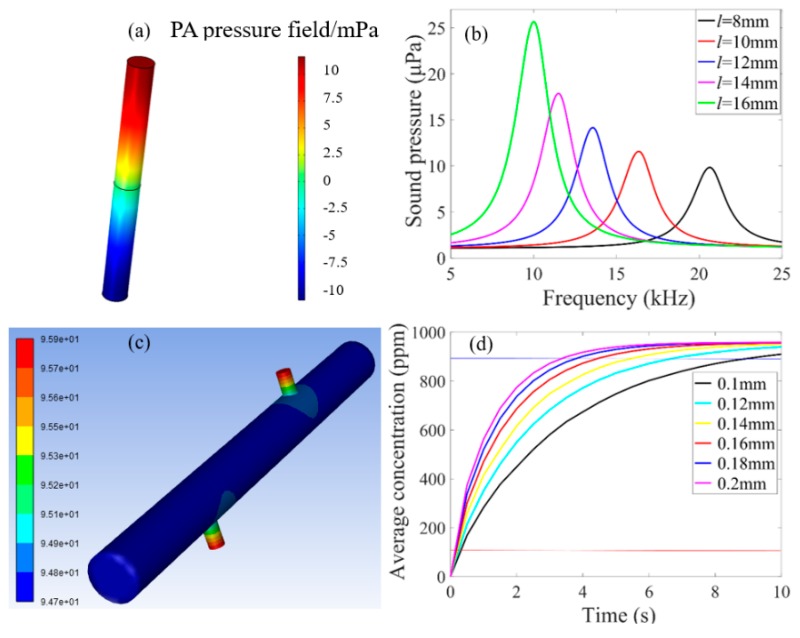
(**a**) Simulated PA field distribution cloud map of the PA sensor with a length of 10 mm at the first-order resonance frequency. (**b**) Simulated amplitude-frequency response of the PA sensor with different cavity lengths. (**c**) Cloud map of simulated gas diffuse concentration in the PA microcavity with 0.15 mm radii of the small holes. (**d**) Simulated variation of the average concentration in the PA microcavity over time with different radii of the small holes.

A 3D finite element model was constructed by using COMSOL Multiphysics [25,26] to evaluate the amplitude-frequency response of the PA sensor. The temperature parameter used for COMSOL simulation is 20 °C. Figure 2a shows the PA field distributions at the first-order resonance frequency of the PA sensing head. The antinodes of the acoustic standing wave are located in the dark (red or blue) areas of the two endfaces of the PA cavity, where the maximum PA pressure exists. Meanwhile, the node of the acoustic standing wave is located in the middle of the PA cavity. The boundary layers on the inner wall of the PA tube have been taken into account in the finite element simulation model. The boundary layer will cause the standing wave to lose in the energy accumulation process, which will reduce the Q-factor. It also has a small effect on the resonance frequency [27]. Figure 2b shows the simulated amplitude-frequency response of the PA sensor with different cavity lengths (*l* = 8, 10, 12, 14, 16 mm). The plotted function corresponds to the magnitude of *P*_PA_. A longer PA cavity length has a lower first-order resonance frequency and is beneficial to increase the PA response at the resonance frequency. However, in order to reduce the gas response time, the cavity should be small. Considering that the highest response frequency of the MEMS microphone was 20 kHz, the length of the stainless-steel PA cavity was chosen to be 10 mm. The corresponding cavity volume of the sensor was calculated to be only 7.9 μL. When the sound pressure is highest, the first-order resonance frequency is 16.344 kHz. 

The simulation of the gas diffusion process in the PA microcavity was employed by the finite element analysis software of ANSYS Fluent [28]. The response time of the sensor was analyzed when the radii of the two small holes were changed. Figure 2c shows the cloud map of simulated gas diffuse concentration in the PA microcavity. The radii of the small holes are 0.15 mm. By changing the radii of the holes in the model, the average gas concentration in the PA microcavity as a function of time was simulated, as shown in Figure 2d. The larger the radius of the hole, the shorter the response time. However, a big hole weakens the isolation of the external sound field. The radii of two small holes were chosen to be 0.14 mm in order to increase the gas concentration from 10% to 90% within 5 s.

**Figure 3:**
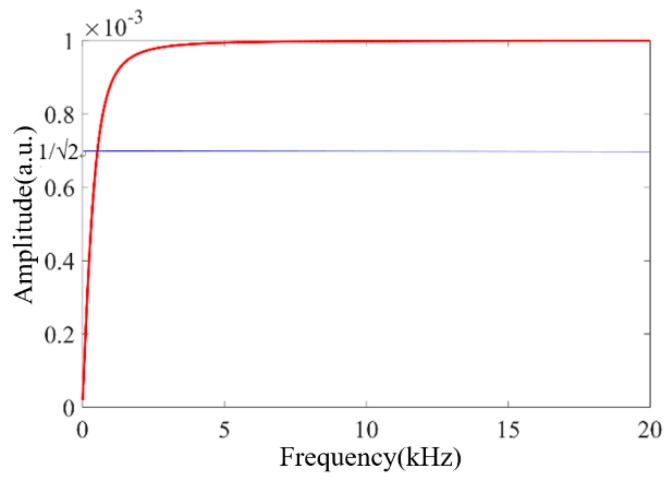
Calculated amplitude of *H*(*τ*_2_) as a function of frequency.

The effect of air flow and heat flow caused by the small holes on the PA response can be regarded as a high-pass filter [29,30], as shown in Equation (3). When the length of the stainless-steel capillary is 10 mm, the inner radius of the capillary is 0.5 mm and the radii of two small holes are 0.14 mm, the relationship between the amplitude of *H*(*τ*_2_) and the frequency is calculated, as shown in Figure 3. The cutoff frequency of *H*(*τ*_2_) is about 550 Hz, which is well below the first-order resonance frequency (16.344 kHz) of the PA cavity. If the sensor operates at the resonance frequency, the generated PA pressure wave is almost confined within the microcavity. At the same time, the external high-frequency disturbing sound field can also be effectively isolated. As a result, combined with the narrow-band signal detection technology, the error of the interference sound field on the PA gas concentration measurement can be greatly reduced.

### 2.2. Experimental Setup

**Figure 4:**
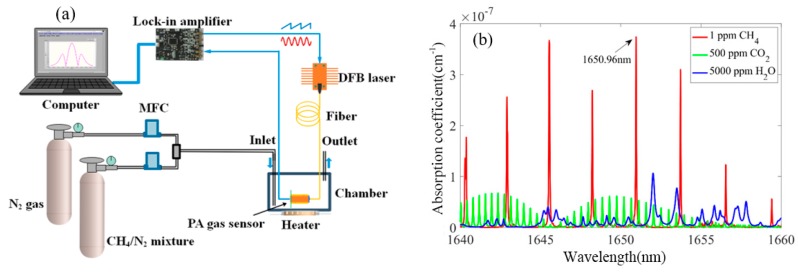
(**a**) Schematic structure of the test system for diffusing gas detection. MFC: mass flow controller; PA: photoacoustic; DFB: distributed feedback. (**b**) Absorption coefficients of 1 ppm CH_4_ and interfering gases in the spectral range from 1640 nm to 1660 nm.

The structure of the test system for diffused CH_4_ gas detection is schematically shown in Figure 4a. To eliminate the interference caused by the 1*f* background generated by the light absorption of wavelength independent sources, such as the tube wall, the 2*f*-WMS technique [10,31] was applied. A distributed feedback (DFB) laser was modulated by a combined signal generated by the addition of a sine wave and a sawtooth wave to generate 2*f*-WMS signal. The working temperature of the laser was 24 °C. In addition, the sinusoidal modulation amplitude was set to 5.1 mA in order to maximize the 2*f*-signal [32]. A homemade lock-in amplifier was designed to provide sinusoidal/sawtooth excitation to the DFB laser and detect the 2*f* component of the PA signal. Since the lock-in amplifier is equivalent to a high-Q bandpass filter, noise can be greatly suppressed. The laser light was injected into the PA sensor through an optical fiber. The lock-in amplifier extracted the 2*f* component of the PA signal. The miniature PA sensor was placed in a chamber of ~150 mL volume. To study the influence of temperature, we used a heater to adjust the temperature of the aluminum chamber. The gas configuration system consisted of a CH_4_/N_2_ gas mixture, a pure N_2_ gas and two mass flow controllers (MFCs). Different gas concentrations of the test gas were obtained by controlling the flow ratio of the two MFCs. The outlet of the chamber was opened during gas injection. The distinguishing features of the sensor are low power and low cost. The total power consumption is approximately 1.5 W. Since there are no valves and pumps that consume a lot of power, the power consumption can thus be reduced by about 2 W [33]. The power consumption is comparable to the diffusion-type TDLAS gas sensor, which can also avoid the need of a gas valve and pump [34]. The total hardware cost of the PA gas sensor is only about $400. For multi-point gas leak monitoring, a DFB laser can provide a PA excitation source to multiple probes in order to further reduce the power consumption and the cost of a single point measurement.

In consideration of the low cost of near-infrared lasers, the preferred characteristic wavelength for detecting CH_4_ is selected in the near-infrared region. According to the HITRAN2012 database [35], the absorption coefficients of 1 ppm CH_4_, 500 ppm CO_2_ and 5000 ppm H_2_O are plotted in Figure 4b in the spectral range from 1640 nm to 1660 nm. The absorption line of CH_4_ at the wavelength of 1650.96 nm has a higher absorption coefficient and lower interference of H_2_O and CO_2_. Even if the water vapor concentration is as high as 50,000 ppm, the interference to the CH_4_ concentration measurement is still less than 1 ppm. The output power of the DFB laser was ~9 mW at a driving current of 95 mA. Since both the injection current and temperature affect the laser wavelength, the bias current was regulated by a signal generator, and the temperature was thermostatically controlled by a thermoelectric cooler (TEC). 

## 3. Experimental Results and Discussion

### 3.1. Amplitude-Frequency Response

**Figure 5:**
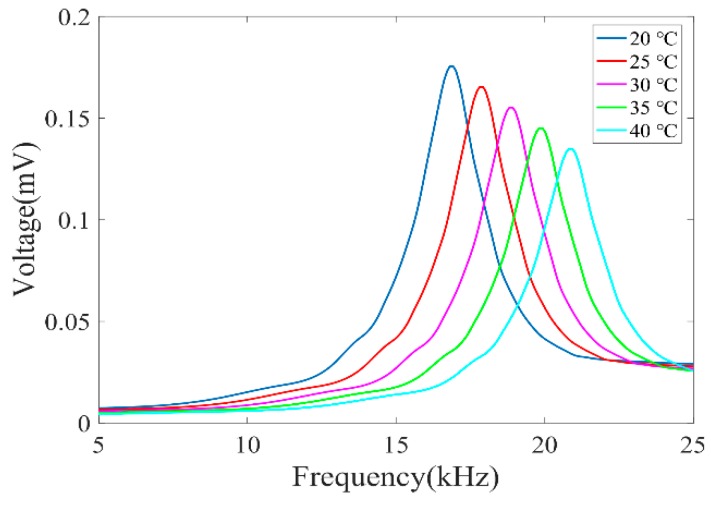
Measured amplitude-frequency response of the PA microcavity gas sensor filled with 1000 ppm CH_4_/N_2_ gas mixture under different temperatures.

We measured the amplitude-frequency response of the PA microcavity gas sensor with different temperatures of 20 °C, 25 °C, 30 °C, 35 °C, and 40 °C. The chamber was filled with CH_4_/N_2_ gas mixture at a concentration of 1000 ppm. The lock-in integration time was 1 s. By stabilizing the bias current at 94.9 mA, the wavelength of the DFB laser was fixed at 1650.96 nm. We scanned the sinusoidal current modulation frequency from 2.5 to 12.5 kHz, and recorded the 2*f*-WMS signal from 5 to 25 kHz, as shown in Figure 5. With the increase of the temperature, the resonance frequency increased. This is because the sound velocity increases with the increase of temperature. Meanwhile, it indicates that the signal value at the resonance frequency decreases as the temperature increases. This is mainly because $H(\tau_{1})$ decreases as the frequency increases, when the resonance frequency increases. In order to achieve a high-precision concentration measurement, a method of peak finding after frequency scanning and temperature compensation can be used. When the temperature is 20 °C, the sensor has the highest response at 16.62 kHz, which is in good agreement with the theoretical result of 16.344 kHz. In addition, the calculated quality factor is 9.51. In the following experiment, we set the sinusoidal current modulation frequency to be 8.312 kHz in order to maximize the 2*f* PA pressure signal. The gas detection experiment was carried out under the conditions of an ambient temperature of ~21 °C and a relative humidity of ~17%.

### 3.2. Concentration Response

**Figure 6:**
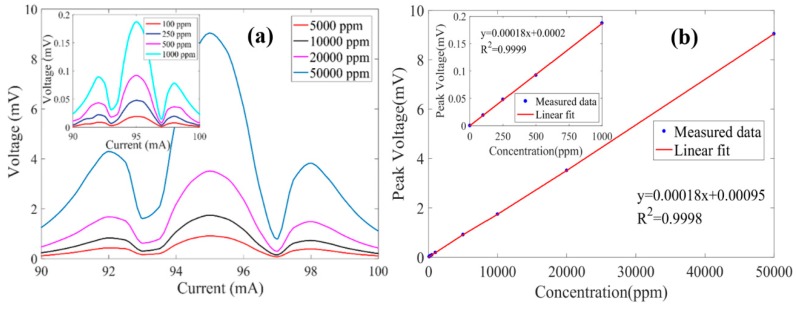
(**a**) Measured 2*f*-WMS signals with different concentrations of CH_4_. (**b**) Relationship between peak value and gas concentration.

In order to measure 2*f*-WMS signals of different CH_4_ concentrations near the wavelength of 1650.96 nm, the bias current of the laser was increased from 90 mA to 100 mA. The root mean square (RMS) value of the PA signal was measured by the lock-in amplifier. The lock-in integration time was set to 1 s. Figure 6a shows the 2*f*-WMS signal of CH_4_ at concentrations of 100 ppm, 250 ppm, 500 ppm, 1000 ppm, 5000 ppm, 10,000 ppm, 20,000 ppm, and 50,000 ppm, respectively. Since the laser wavelength at the current of 94.9 mA is 1650.96 nm, which corresponds to the gas absorption peak, the 2*f* PA signal value is the largest. In practical applications, due to factors such as ambient temperature, the laser wavelength at a fixed current would slightly drift. Therefore, the laser current was scanned, and then the peak value of the measured 2*f*-WMS signal was sought. The relationship between the measured peak of the 2*f*-WMS signal and the concentration of CH_4_ is plotted in Figure 6b. By using a linear fit, the estimated concentration responsivity was obtained at 0.18 μV/ppm for CH_4_ gas detection. In addition, the calculated R-squared value was 0.9998, which shows that the PA microcavity gas sensor had a good linear response to CH_4_ concentration levels below 50,000 ppm.

### 3.3. Response Time and Recovery Time

**Figure 7:**
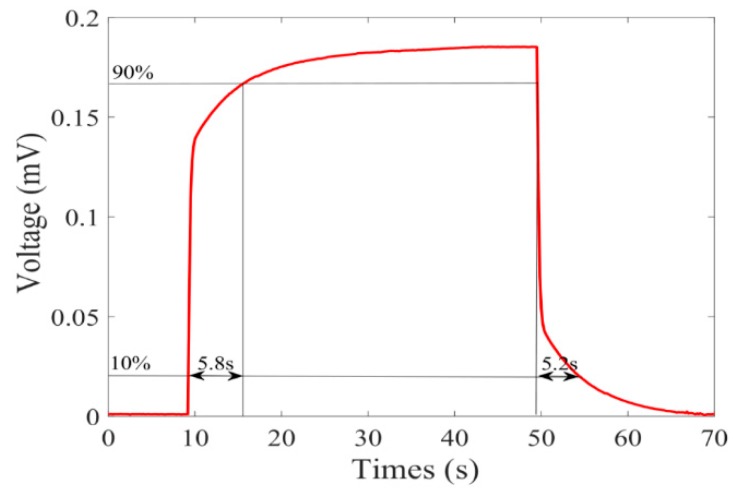
Continuous measurement result of 1000 ppm CH_4_.

The response and recovery time of the PA microcavity gas sensor were measured. A CH_4_/N_2_ gas mixture with a concentration of 1000 ppm was injected into the gas chamber. The lock-in integration time was 1 s. After about 20 s, pure N_2_ was then injected. The measured amplitude of the PA signal is shown in Figure 7 with a 1-s lock-in integral time. The measured voltage from 10% to 90% required a response time of 5.8 s, and a reduction of 90% to 10% required a recovery time of 5.2 s. Since the actual size of the sensor may be different from the designed parameters, the test result of response time was slightly longer than the simulation result. In addition, the stable value of 185.2 μV was basically consistent with the result of peak signal of 187.6 μV shown in Figure 6a.

### 3.4. Detection Limit

**Figure 8:**
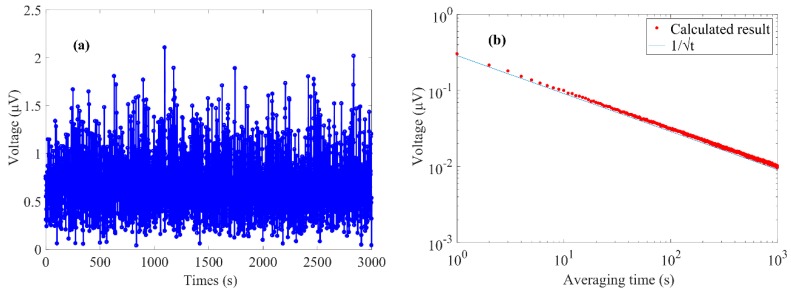
(**a**) PA signal with a 1-s lock-in integral time when the chamber was filled with ambient air. (**b**) Allan–Werle deviation analysis result.

To determine the MDL of the PA microcavity gas sensor, the signal was measured with the gas chamber filled with ambient air. The amplitude of the PA signal was recorded with a 1-s lock-in integral time, as shown in Figure 8a. The calculated standard deviation (1σ) of the measurement result of ambient air was 0.31 μV. The corresponding MDL was obtained at 1.7 ppm according to the concentration responsivity of 0.18 μV/ppm. The calculated minimum detectable absorption coefficient was 6.6 × 10^−7^ cm^−1^. Meanwhile, the calculated normalized noise equivalent absorption (NNEA) coefficient was 1.2 × 10^−8^ W·cm^−1^·Hz^−1/2^. An Allan–Werle deviation analysis [11,36,37,38,39] was performed, and the result is shown in Figure 8b. The analyzed result almost follows a dependence of 1/√t, which indicates that the PA microcavity gas sensor is affected by Gaussian noise during the 3000-s test time. The deviation is 0.03 μV with a 100-s averaging time, and the MDL can be improved to 0.17 ppm.

### 3.5. Discussion

For practical application, the influence of water vapor induced vibration-rotation (V-T) relaxation should be considered. Schilt [40] and Wu [41] have studied this problem in detail, successively. In addition, the calibration method has been presented by using an additional near-infrared laser to measure the concentration of H_2_O. In our future work, we will firstly find out the relationship between the amplitude of the PA signal and the water vapor concentration. Subsequently, the influence of the V-T relaxation can be calibrated by the vapor concentration, which is measured by a temperature/humidity sensor installed on the circuit.

## 4. Conclusions

In summary, a PA microcavity sensor was proposed and developed for gas leak detection with the advantages of high sensitivity, low power, and low cost. The designed PA gas sensor mainly consisted of a stainless-steel capillary and a MEMS microphone. The leaking gas could diffuse into the stainless-steel PA cavity from two small holes opened by the laser marker on the side wall of the capillary. The volume of the PA microcavity was only 7.9 μL. The optimized radius of the two holes was 0.14 mm. A DFB laser operating at 1650.96 nm was used to detect CH_4_. A good linearity response of the PA microcavity sensor to CH_4_ concentration less than 50000 ppm was demonstrated with a responsivity of 0.18 μV/ppm. The response time and recovery time were measured to be 5.8 and 5.2 s, respectively. In addition, the MDL and the minimum detectable absorption coefficient of the designed PA microcavity sensor was achieved at 1.7 ppm and 6.6 × 10^−7^ cm^−1^ with a 1-s lock-in integral time, respectively. The calculated NNEA coefficient was 1.2 × 10^−8^ W·cm^−1^·Hz^−1/2^. The designed PA gas sensor has the advantages of a small volume, simple structure, low cost, high sensitivity, fast response and recovery time, and no need for gas valves and pumps. By changing the laser source, it is possible to detect various trace diffused gases such as NH_3_, H_2_S, C_2_H_2_ and C_2_H_4_.
